# Calculation of Linear and Non-linear Electric Response Properties of Systems in Aqueous Solution: A Polarizable Quantum/Classical Approach with Quantum Repulsion Effects

**DOI:** 10.1021/acs.jctc.0c00674

**Published:** 2020-10-15

**Authors:** Gioia Marrazzini, Tommaso Giovannini, Franco Egidi, Chiara Cappelli

**Affiliations:** † Scuola Normale Superiore, Piazza dei Cavalieri 7, Pisa 56126, Italy; ‡ Department of Chemistry, Norwegian University of Science and Technology, Trondheim 7491, Norway

## Abstract

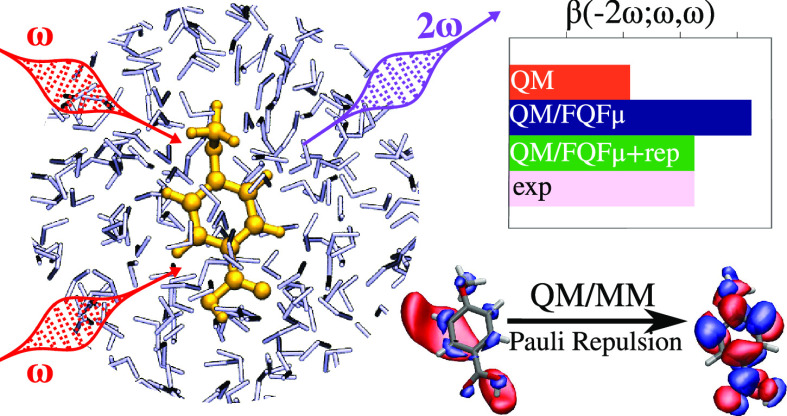

We present a computational study of polarizabilities and hyperpolarizabilities of organic molecules in aqueous solutions, focusing on solute–water interactions and the way they affect a molecule’s linear and non-linear electric response properties. We employ a polarizable quantum mechanics/molecular mechanics (QM/MM) computational model that treats the solute at the QM level while the solvent is treated classically using a force field that includes polarizable charges and dipoles, which dynamically respond to the solute’s quantum-mechanical electron density. Quantum confinement effects are also treated by means of a recently implemented method that endows solvent molecules with a parametric electron density, which exerts Pauli repulsion forces upon the solute. By applying the method to a set of aromatic molecules in solution we show that, for both polarizabilities and first hyperpolarizabilities, observed solution values are the result of a delicate balance between electrostatics, hydrogen-bonding, and non-electrostatic solute solvent interactions.

## Introduction

The investigation of non-linear optical properties of molecular systems has for long been of particular interest owing to the peculiar optical behavior of materials that possess a high non-linear response, which have found applications in fields such as signal processing and telecommunications.^1^ In parallel with experimental advances, a significant amount of effort has been devoted to the development of computational protocols to aid in both predicting and rationalizing the non-linear optical response a molecule or material in the condensed phase.

In fact, the problem of accurately simulating electric response properties of molecular systems in solution has been the object of many studies over the years, with research effort focusing on increasing the accuracy of the quantum mechanics (QM) methods employed for the simulation of the light–matter interaction, which is at the origin of the response, as well as investigating different strategies to incorporate environmental effects into the calculation, particularly in the case of molecules in liquid solutions.^2−9^

Ab initio calculations typically rely on a choice of a model to treat electron-correlation effects coupled to a suitable basis set, and different levels of theory have been explored in the literature.^10−24^ The electronic component alone is sometimes not enough to properly reproduce both the linear and non-linear optical response of molecules, and vibrational effects can be quite relevant. Several studies have delved into this problem and offered computationally efficient solutions.^5,25−27^ When it comes to the modeling of environmental properties, the literature has mostly focused on ways to model the purely electrostatic component of the solute–solvent interaction, both to produce general solvation models, and as it pertains to the calculation of linear and non-linear optical properties themselves.^28−35^

Because electrostatic interactions are long-range, an atomistic description of the solvent that properly accounts for the effect upon the solute has to include a large number of molecules. This fact, combined with the large configurational space of the solute–solvent system that should be sampled, makes a fully quantum-mechanical description computationally prohibitive. Mixed quantum-classical focused models that treat the solute quantum-mechanically while resorting to a classical description of the solvent, which can be treated as either a continuum or by preserving the atomistic detail and describing the latter using molecular mechanics (MM) models, are a suitable alternative.^36−39^ In the most basic formulation, QM/MM models only account for the electrostatic solute–solvent interaction, modeling the solvent by means of fixed charges.^37^ However, solvent polarization effects are crucial, especially if one is interested in linear and non-linear optical properties,^40−45^ because otherwise the solvent remains insensitive to the polarization effects induced upon the molecule by the probing electric field. Polarizable embedding methods establish a mutual polarization between the QM solute and its environment, and the solute–solvent interactions directly affect the former’s response properties.^41−43,46−49^

In recent years, we have implemented a polarizable QM/MM method that endows solvent atoms with charges (FQ) and possibly dipoles (Fμ) that are allowed to fluctuate in response to the solute’s electrostatic potential.^42,46,50,51^ We have shown how the model can have tremendous success in describing a wide array of spectroscopic properties of molecules in water, a highly polar solvent that can form hydrogen bonds with the solute. The properties we have studied include Raman spectroscopy and Raman optical activity,^51,53^ electronic and vibrational absorption and circular dichroism,^53−56^ two-photon absorption,^57^ optical rotation,^58,59^ and electronic paramagnetic resonance.^60^ The model describes electrostatic interactions through its fluctuating charges and dipoles that dynamically respond to changes in the solute’s electronic density and has recently been extended to the treatment of non-electrostatic dispersion and repulsion effects.^60−62^ These effects can be critical in determining linear and non-linear electronic properties of a system.^63^ The quantum repulsion exerted by the solvent upon the solute’s electron density, in particular, has the effect of confining it within the cavity occupied by the solute and is therefore expected to reduce the latter’s polarizability and hyperpolarizability. Commonly employed solvation models, including the popular polarizable continuum model (PCM)^36^ only account for solute–solvent electrostatics and therefore are missing any confinement effect due to repulsion forces. Note that alternative embedding methods that treat some solvent molecules quantum-mechanically can include repulsion effects naturally through the quantum treatment. These methods often include a classical solvent layer, resulting in a QM/QM/MM paradigm. The QM/FQ and related paradigms, however, find their strength in being “focused” models, where only the properties of the solute and solute–solvent interactions are accurately treated, while the properties of the solvent itself are not of interest, which helps limit the computational cost.

For these reasons, electrostatic, polarization, and quantum repulsion effects are all expected to be particularly relevant in the case of non-linear electric response properties, and it is therefore worth exploring the importance of these effects on model systems, both to confirm these intuitions and highlight the shortcomings in standard calculations based on environmental models, which often neglect one or more of these effects, as well as the magnitude of the errors that would be committed. To this end, we show how different solvation forces contribute to the overall linear and non-linear optical response on a set of six aromatic molecules in solution by employing different electrostatic models based on the QM/FQ(Fμ) paradigm, further enriched by the inclusion of repulsion forces. This is the first time this solvation model is applied to non-linear optical response properties. We show that repulsion forces can indeed be just as important, if not even more so, to the determination of a solute’s (hyper)polarizability as electrostatic interactions, even for a solvent as polar as water. In the next section, the theoretical model is briefly recalled in its various components followed by a description of the computational protocol and the analysis of the results. A summary of the work and future perspectives conclude the manuscript.

## Theoretical Background

Molecular polarizabilities and hyperpolarizabilities can be related to the microscopic response of a molecular system to an external electric field **E**(*t*), represented by an induced dipole moment *μ*(*t*):
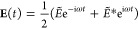
1
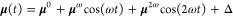
2where ω is the frequency of the monochromatic incident light, and *Ẽ* is the complex constant amplitude of the field. The Fourier amplitude in eq 2 can be rewritten as a Taylor expansion with respect to the external electric field.^64^ In particular, second harmonic generation (SHG), i.e., the generation of a photon at 2*ω* as a result of the interaction with an incident ω photon reads:^64^
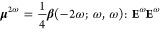
3

The first hyperpolarizability *β* is a third-rank tensor that can be described by a 3 × 3 × 3 matrix, whose 27 components are not independent and can be reduced assuming Kleinman’s symmetry.^65^

By exploiting the response theory formalism, the first-order hyperpolarizability  can be calculated as^66,67^
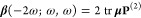
4where *μ* is the electric dipole moment integral matrix and **P**^(2)^ is the second-order density matrix. A generic second-order density matrix is obtained by solving perturbed equations up to the second order; however, when only one dynamic perturbation is involved, it is possible to avoid the solution of the second-order coupled perturbed equations by using an iterative procedure to reconstruct the density matrix.^66−68^

Hyperpolarizabilities produced by QM calculations are three-indices tensor quantities. Any meaningful comparison between calculated and experimental data must refer to certain rotational invariants that can be obtained from the full tensor, depending on the specifics of the experimental setup one wishes to reproduce. In this work, we compare our results with those obtained from hyper-Rayleigh scattering (HRS)^69,70^ experiments presented in ref (71). In that work, a comparison between computed and experimental results was done by referring to the following quantity:
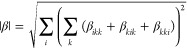
5

Therefore, we refer to the same quantity for the sake of comparison between calculated and experimental data, as was also done in a previous work.^72^ However, it is worth noticing that alternative definitions for HRS values have been proposed in the literature, giving computed results directly comparable with experimental data.^20,70,73^

In the following, within tables and figures, we use the notation *β*( – 2*ω*; *ω*, *ω*) in order to emphasize the particular type of frequency dependence; however, note that the presented values always refer to eq 5.

Molecules in solution interact dynamically with the solvent through both electrostatic and non-electrostatic forces. The solute–solvent interaction energy depends on the solute’s electronic density, which is affected by the probing electromagnetic field. Therefore, an embedding model that seeks to capture solvation effects upon a measured linear and non-linear electric response property should take the dynamical aspects of the mutual solute–solvent interaction into account. In this work, we employ the fully atomistic QM/FQ and QM/FQFμ models to describe the electrostatic interactions between the solute and solvent, while resorting a recently implemented model to account for Pauli repulsion effects, the details of which are recalled in the following section.

### Solvation Model

As explained above, in this work, we are adopting a multiscale QM/MM approach to describe solvent effects on a QM solute. In particular, the interaction energy *E*_QM/MM_^int^ between the QM and MM layers is formulated as
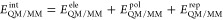
6where *E*_QM/MM_^ele^ and *E*_QM/MM_^pol^ are the electrostatic and polarization contributions, respectively, whereas the last term *E*_QM/MM_^rep^ is the Pauli repulsion, which acts a density confinement. It is worth remarking that we are not including any QM/MM dispersion interaction term. Because of the nature of QM/FQ being a focused model, by neglecting dispersion effects, the solute electronic density is not allowed to delocalize toward the solvent. It is however worth remarking that dispersion plays only a minor role in aqueous solutions, although eq 6 can be extended to account for such an interaction,^47,60,62,74^ though of course it may be quite relevant for other solvents.

In order to treat the electrostatic QM/MM coupling, two different polarizable QM/MM approaches were considered, namely, QM/FQ^42,46,52,53,57,59^ and QM/FQFμ.^50,51,75^ In the former, each atom of the MM portion is endowed with a charge (*q*), which can vary in agreement with the electronegativity equalization principle (EEP), i.e., a charge flow occurs between two atoms at a different chemical potential. FQ force field is defined in terms of two atomic parameters, namely, electronegativity (*χ*) and chemical hardness (*η*). The latter (QM/FQFμ) is instead a pragmatical extension of FQ, in which fluctuating atomic dipoles (*μ*) and fluctuating atomic charges (*q*) are associated to each MM atom.^50^ Charges values are defined by the same charge equilibration as FQ, but their values depend also on the interaction with dipoles. The peculiarity of FQFμ stands in the fact that both FQ’s and Fμ’s vary according to the electric potential and electric field.

In order to model Pauli repulsion, an approach recently proposed by some of the present authors is used.^60−62^ There, each MM molecule is endowed with a set of s-type Gaussian functions, which mimic the presence of a QM density in the MM portion (Pauli repulsion interaction is a purely quantum effect due to Pauli principle). In our approach, the repulsion energy term is written as the opposite of an exchange integral:^63,76,77^
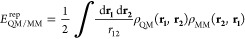
7

In order to define the density *ρ*_MM_ , we localize fictitious valence electron pairs for MM molecules in bond and lone pair regions and represent them by s-Gaussian-type functions. The expression for *ρ*_MM_ becomes
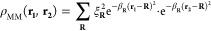
8where **R** runs over the centers of the Gaussian functions used to represent the fictitious MM electrons. The *β* and *ξ* parameters are generally different for lone pairs or bond pairs, their values being adjusted to the specific kind of environment (MM portion) to be modeled. See ref (61) for their definition in the case of the water molecule. By substituting eq 8 in eq 7, the QM/MM repulsion energy reads
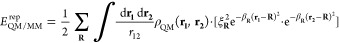
9

It is worth noticing that, in this formalism, QM/MM Pauli repulsion energy is calculated as a two-electron integral. Equation 9 is general enough to hold for any kind of MM environment (solvents, proteins, surfaces, etc.). The nature of the external environments is specified by defining the number of different electron-pair types and the corresponding *β* and *ξ* parameters in eq 8. Finally, the formalism is general so that it can be coupled to any kind of QM/MM approach.

All of the components of this solvation model require a specific parametrization.

## Computational Details

**Figure 1:**
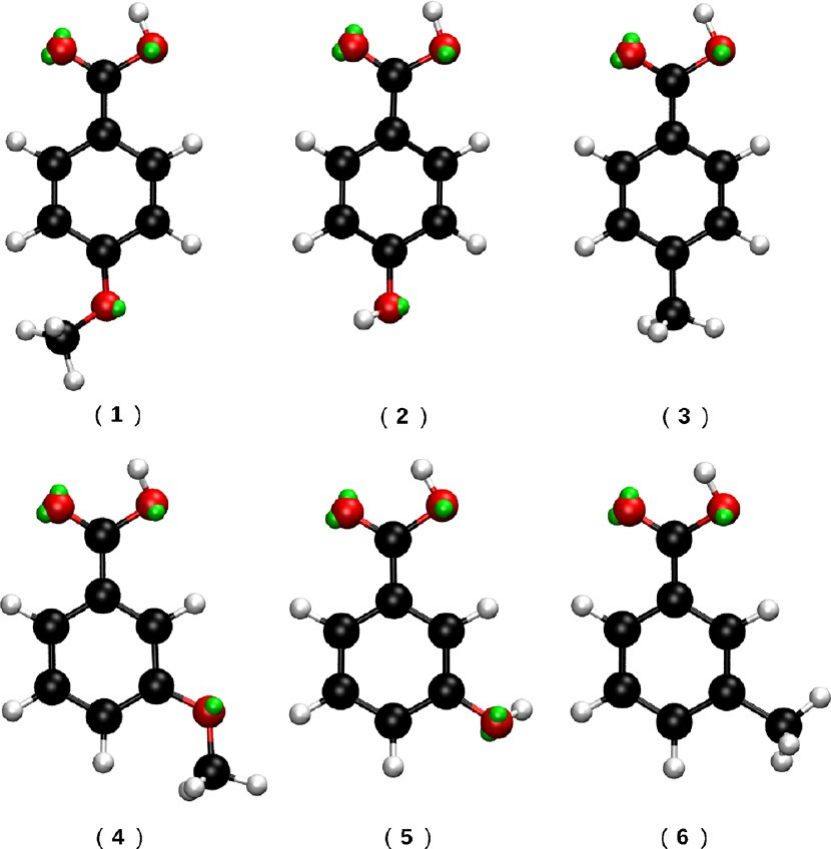
Structures of the molecules studied. The green spheres depicted close to the oxygen atoms represent the virtual sites (VS, *vide infra*).

For this work, we have selected six organic molecules (Figure 1) from ref (72), for which experimental measurements of their first hyperpolarizability values in aqueous solutions exist.^71^ All QM and QM/MM calculations were performed using a locally modified version of Gaussian16 computational chemistry package^78^ and employed the B3LYP,^79−81^ CAM-B3LYP,^82^ and M06-2X^83^ density functionals in combination with the 6-311++G(d,p) basis set. Polarizable QM/MM calculations were performed with the fluctuating charge model (FQ)^42,46,84−86^ with and without fluctuating dipoles (FQFμ).^50^ QM/FQ calculations were performed using two distinct parametrizations, the one by Rick et al.,^84−86^ which we here denote as FQ*^a^*, and the one by Giovannini et al.,^60^ denoted as FQ*^b^*. Hyperpolarizabilities are reported in esu.^87^ In order to compute hyperpolarizabilities with the QM/MM methodology described above, we followed a multistep procedure, which is here summarized:

1. Geometry optimization of the solute molecules. The structure of each system was optimized using the CAM-B3LYP density functional and by including solvent effects by means of the PCM.^88−90^
2. Calculation of atomic charges and definition of virtual sites. From the same CAM-B3LYP/PCM calculations on the optimized structures, we obtained the RESP atomic charges^91−93^ and locations for the virtual sites (VS), which model the presence of non-bonding electron pairs. VS have a fixed position with respect to generating atoms and allow us to refine the description of hydrogen-bonding interactions. The positions were obtained by evaluating the centroids of Boys orbitals.^94,95^
3. Classical MD simulations in aqueous solutions. Each solute molecule was placed in a cubic box and then surrounded by water molecules under periodic boundary conditions (PBC). To sample the solute–solvent configuration space, a classical MD simulation on each system was run as detailed in ref (72).
4. Extraction of snapshots from the MD simulation. From each MD run, a total of 200 snapshots was extracted to be used in the QM/MM calculations for each system. For each snapshot, a solute-centered sphere with radius of 15 Å of explicit water molecules was cut.
5. Polarizable QM/MM calculations. The QM/MM calculations of static and dynamic polarizabilities and hyperpolarizabilities were performed on the full set of structures extracted from the MD. The results obtained for each spherical snapshot were extracted and averaged to produce the final value.

## Numerical Results

### Effect of Repulsion on the MOs

In this section, we wish to provide a more in-depth analysis of the effect of quantum repulsion and how it enters the computational results. As stated earlier, the addition of quantum repulsion affects the molecular orbitals (MO) of the system. This change then propagates to response equations and therefore computed electric response properties. Changes in the MOs caused by repulsion can be appreciated by plotting the matrix **J** that relates one set of MOs into the other:
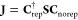
10where **C**_rep_ is the MO coefficient matrix calculated at the QM/FQFμ level with Pauli repulsion, **S** is the atomic orbital overlap matrix, and **C**_norep_ is the MO matrix calculated at the same level without Pauli repulsion.

**Figure 2:**
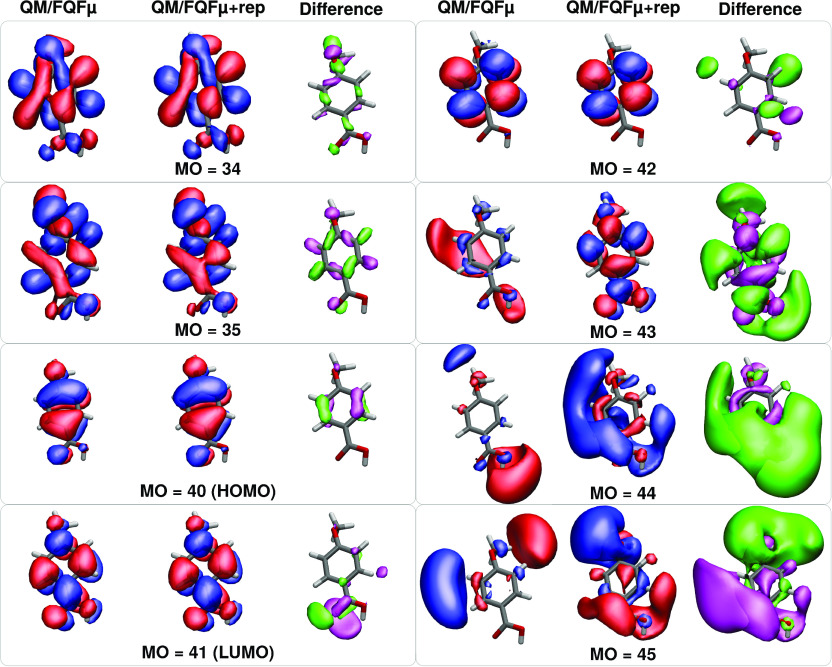
Selected molecule **1** molecular orbitals for a randomly chosen snapshot extracted from the MD simulation. QM/FQFμ and QM/FQFμ+rep orbitals and their difference are depicted.

**Figure 3:**
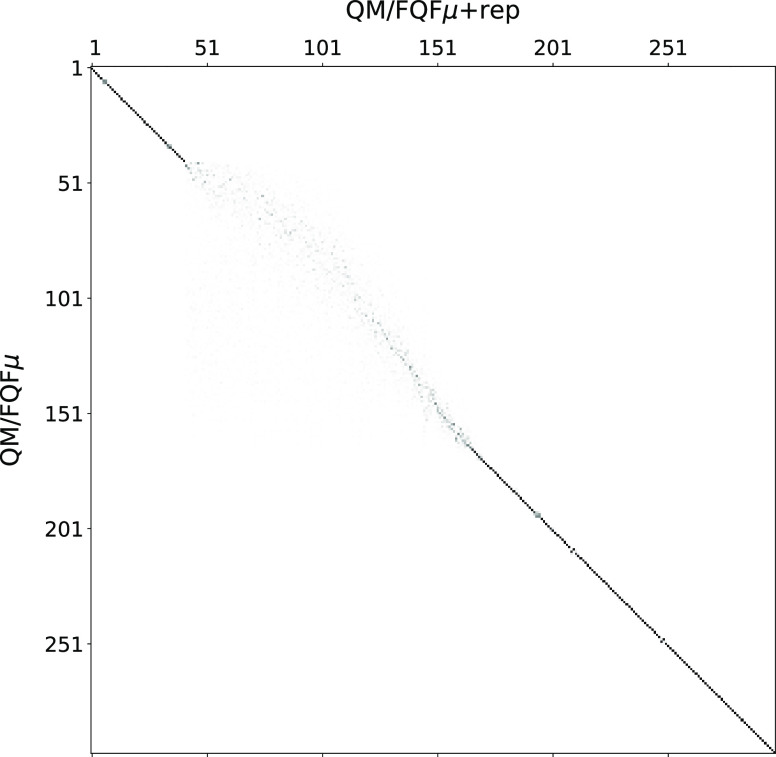
**J** matrix of a randomly snapshot extract from MD simulation (see eq 10)

We performed this analysis for a randomly selected snapshot of the molecule **1**, and the result can be seen in Figure 3 where higher absolute values are represented by a darker square. As expected, occupied orbitals remain mostly unaffected, though this is not true in general (in particular for MO = 34 and MO = 35, which change somewhat, see Figure 2). Many virtual orbitals are instead mixed up, as is evident from Figure 3 and Figure 2. The latter figure shows isovalue plots of selected MOs with and without repulsion as well as the difference in the squared MOs to help visualize the regions of space where changes are most pronounced. In fact, the **J** matrix becomes so sparse in the block involving the first 100 virtual orbitals that it is barely visible in the figure. This is true up to a point, with very high energy orbitals remaining unaltered.

**Figure 4:**
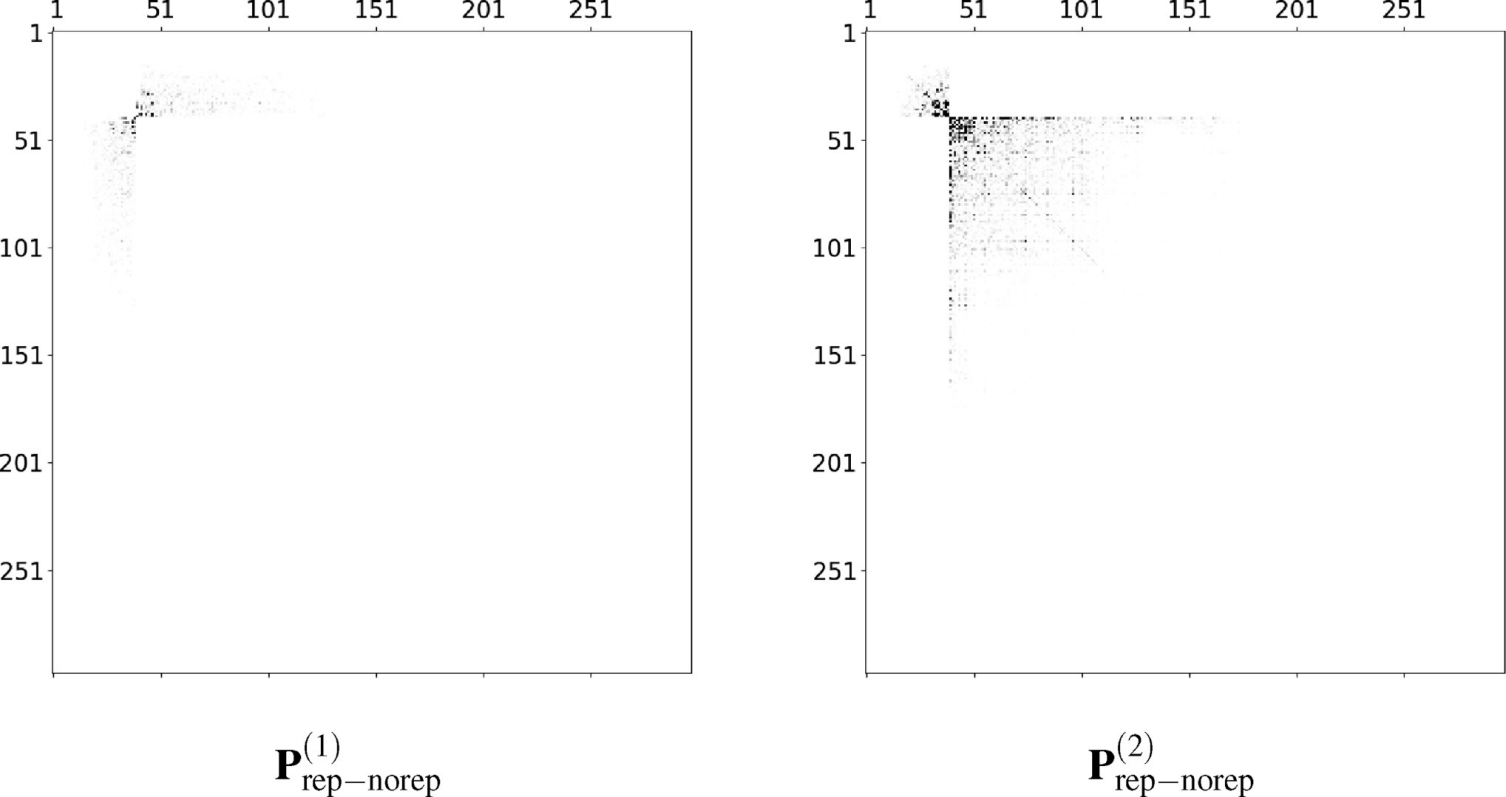
Difference between the density matrix derivatives with and without Pauli repulsion of a randomly snapshot of molecule **1** extracted from MD simulation. The first derivative **P**^(1)^ is on the left panel, and the second derivative **P**^(2)^ is on the right panel. Derivatives are taken with respect to the *z* component of the electric field.

It is worth investigating whether these changes how much these changes actually affect the density derivatives since they are what actually gives the hyperpolarizabilities according to eq 4. Given the large number of components, we only look at derivatives along the *z* component of the electric field. Derivatives with respect to the other components can be found in the Supporting Information. The first-order density derivative **P**^(1)^ (with respect to an electric perturbation along the *z* direction) is non-zero only in the occupied-virtual block. The difference between the two blocks (with and without repulsion) is shown on the left panel in Figure 4. Indeed, while differences are generally negligible, some deviations are observed, particularly in the blocks corresponding to the lowest-energy virtual orbitals that are most affected by repulsion. The same analysis can be carried out for the density second derivative **P**^(2)^, but this time only the occupied-occupied and virtual-virtual blocks are non-zero. Components belonging to higher energy occupied orbitals show a marked difference, while for virtual orbitals, we can draw a similar conclusion as for **P**^(1)^, whereupon only the block involving virtual orbitals that are actually affected by repulsion propagates to density derivatives.

### Polarizability

We begin our investigation by studying the effect of water on static and dynamic polarizabilities.

**Figure 5:**
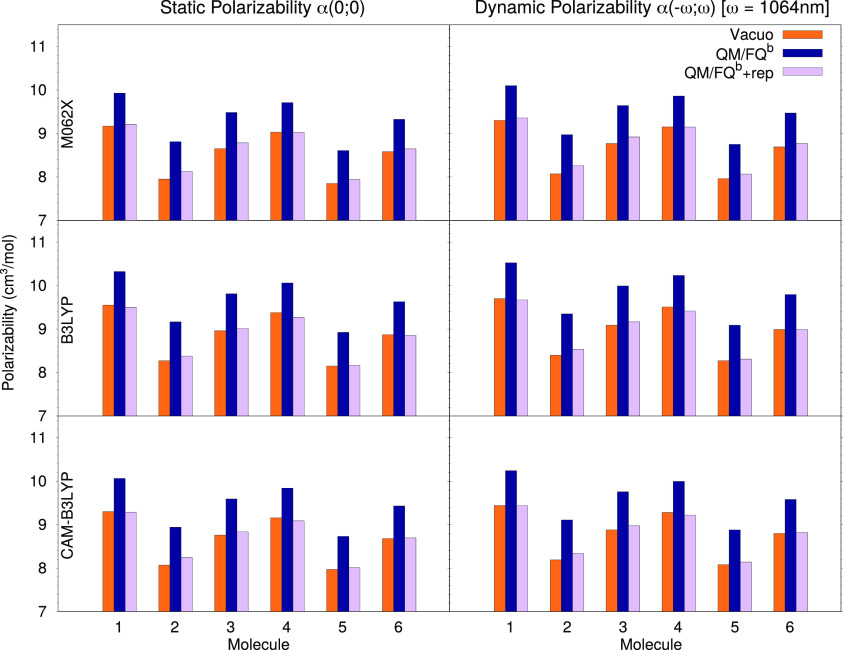
Static (left) and dynamic (right) polarizabilities of molecules **1**–**6** evaluated at 1064 nm in vacuo and in solution (with and without repulsion effects) with three different density functionals: M06-2X (top), B3LYP (middle), and CAM-B3LYP (bottom).

Figure 5 reports the computed values for both the static *α*(0; 0) and dynamic *α*( – *ω*; *ω*) polarizability, evaluated with three different DFT functionals for the isolated and solvated molecules, with and without considering quantum repulsion effects. We start by looking at how a change in the underlying electronic structure model, i.e., the chosen density functional, affects the results, in order to verify that conclusions about solvation effects are consistent and do not depend too much on the functional. It can be immediately seen that the dynamic polarizabilities are substantially higher by about 1.7 units, compared with the static values (see the Supporting Information for tables reporting the numerical values). Solvation electrostatics leads to a significant and uniform increase in the polarizability values for all systems, and the magnitude is rather uniform among the three functionals. It should be noted that the inclusion of repulsion effects into the calculation brings about a significant decrease in the property, by about 8%, and this decrease is actually quite consistent and varies very little among the molecules. Nor are repulsion effects particularly affected by a change in DFT functional, even with the addition of a long-range correction as in CAM-B3LYP. This is not surprising since repulsion effects as modeled in this work directly influence the ground-state density of each system, though they do not directly affect the response functions, for which long-range corrections play their most important role.

**Figure 6:**
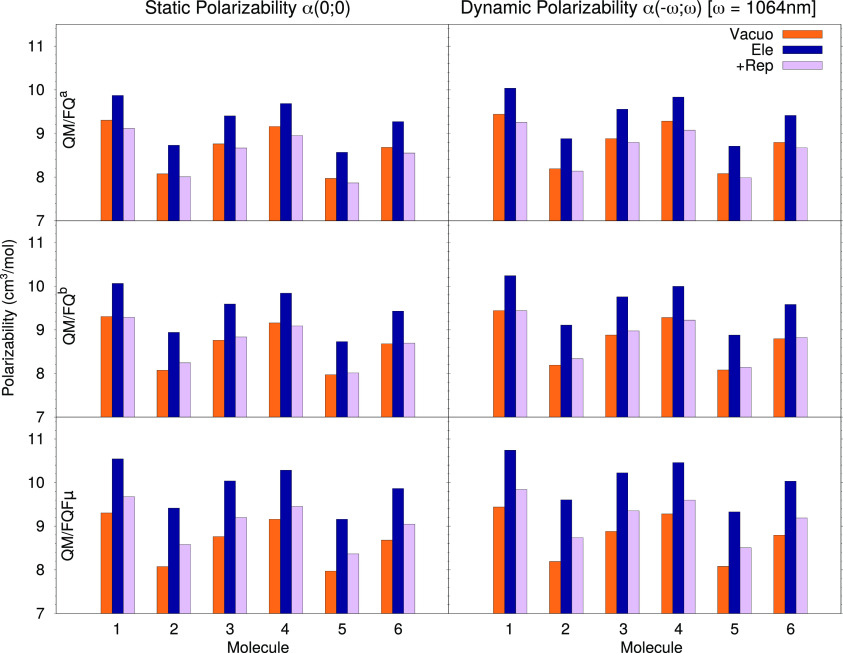
Static (left) and dynamic (right) polarizabilities of molecules **1**–**6** evaluated at 1064 nm in vacuo and in solution (with and without repulsion effects) with three different models for the electrostatic component: FQ*^a^* (top), FQ*^b^* (middle), and FQFμ (bottom).

It is interesting to perform a more in-depth analysis of the different roles of electrostatics and non-electrostatics in determining the polarizability of the solvated systems. As discussed in the Theoretical Background section, there are different sets of parameters to choose from when performing a QM/FQ calculation. Originally, parameters derived by Rick et al.^84^ (hereby denoted as FQ*^a^*) were the first to be developed, though they tend to underestimate the solvent polarization. New parameters specifically designed for QM/FQ calculations were recently adopted,^60^ which allow for a higher solvent polarization. This may not necessarily result in better agreement with experimental values because a higher solvent polarization tends to have an opposite effect compared to the introduction of repulsion forces; therefore, underestimating solvent electrostatics may lead to a favorable error cancellation whenever repulsion effects are neglected. It is therefore interesting to compare values obtained with the different electrostatic models with and without repulsion effects. It is worth reiterating that QM/FQ results are always averages computed over a large set of snapshots obtained from a classical MD, and in order for the results to be reliable, they must be at convergence with respect to the number of snapshots. In the Supporting Information, we show that our results are indeed at convergence. In Figure 6, we present results obtained with the CAM-B3LYP functional only. Indeed, as is evident from the results, going from FQ*^a^* to FQ*^b^*, which leads to an increase in the electrostatics due to the parametrization, does have an opposite effect with respect to repulsion, though the magnitude is not comparable as the FQ*^b^* parameters lead to computed polarizabilities which are about 2 units higher, whereas the reduction due to repulsion effects is significantly stronger. As mentioned in the Theoretical Background section, the basic FQ model can only account for in-plane polarization of solvent molecules; however, out-of-plane solvent polarization may not in principle be disregarded. The FQFμ model overcomes this limitation. Polarizabilities were therefore also evaluated using this electrostatic model with and without repulsion effects. The increase in polarizability that we observe, when going from the FQ*^b^* to the FQFμ values, is of the same order of magnitude as the difference between the FQ*^b^* values and the gas-phase results. Therefore, out-of-plane polarization effects, which can only be taken into account if solvent molecules are endowed with fluctuating dipoles, should not be neglected. It is interesting to note that, if we compare the FQ*^b^*+rep results with the vacuum values (bottom panels in Figure 6), we see that they are very close to the gas-phase values. If the solution values that include all effects were simply compared to those for the isolated molecules, one might erroneously conclude that solvent effects are negligible. Our results show that the role of solvation in determining a system’s polarizability rests on a delicate balance of different effects, none of which can be regarded as negligible; therefore, the use of a solvation model with the capability to include all such effects not only in the description of the system’s ground state but also of its response properties is crucial. It should finally be remarked that solvation models that only treat one of these effects might lead to wrong computed values.

### First Hyperpolarizabilities

We now move to first hyperpolarizabilities, which, being third-order properties, are expected to be much more sensitive to the polarizable environment of the molecule and thus a better probe for the different solvation effects.

As in the case of polarizabilities, gas-phase values are single-point calculations on the optimized structures while QM/FQ results are averages over the structure extracted from the classical MD.

**Figure 7:**
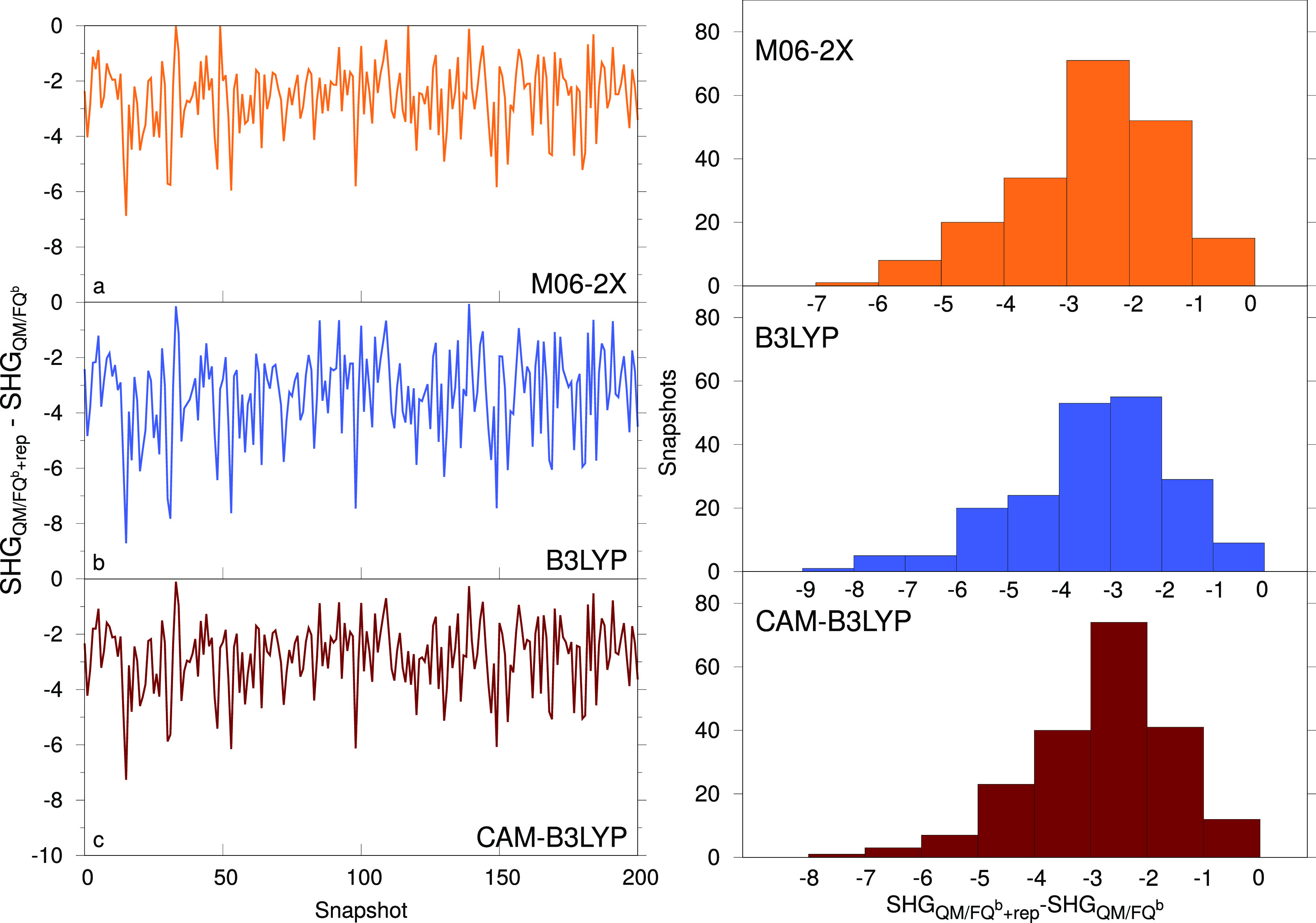
Difference between QM/FQ *β*( – 2*ω*; *ω*, *ω*) (in esu) with and without repulsion for molecule **1** calculated for different snapshots extracted from the MD and for different functionals: CAM-B3LYP, B3LYP, and M906-2X. Values are shown both as they vary across the snapshots (left) and as interval distributions (right).

The solvation effect observed for the average value is the result of changes on each of the extracted MD snapshots. Before commenting on the averages, we therefore analyze the hyperpolarizability values for all snapshots with the different solvation models. Figure 7 reports the difference between the hyperpolarizability values of molecule **1** calculated with the QM/FQ*^b^* model with and without repulsion for all snapshots. Data are also collected into distribution diagrams. The plots show the range of variability in MD time of the calculated property, which depends on the spatial arrangement of the solvent molecules around the solute as well as its instantaneous conformation. Our dynamical atomistic approach to the solvation phenomenon is able to give insight into such a variability, whereas mean-field approaches would instead focus on the average value only. Though the average effect of the hyperpolarizability is of course functional-dependent, it can be readily seen from the plots that they are highly correlated, i.e., given one snapshot if a high or low repulsion effect is obtained for one functional, a similar result will be observed when using the other two. One thing that stands out is that the effect of repulsion is very dishomogeneous across the snapshots, where some have almost no effect and others presenting a decrease in hyperpolarizability that is almost as high as the average value of the property itself.

**Figure 8:**
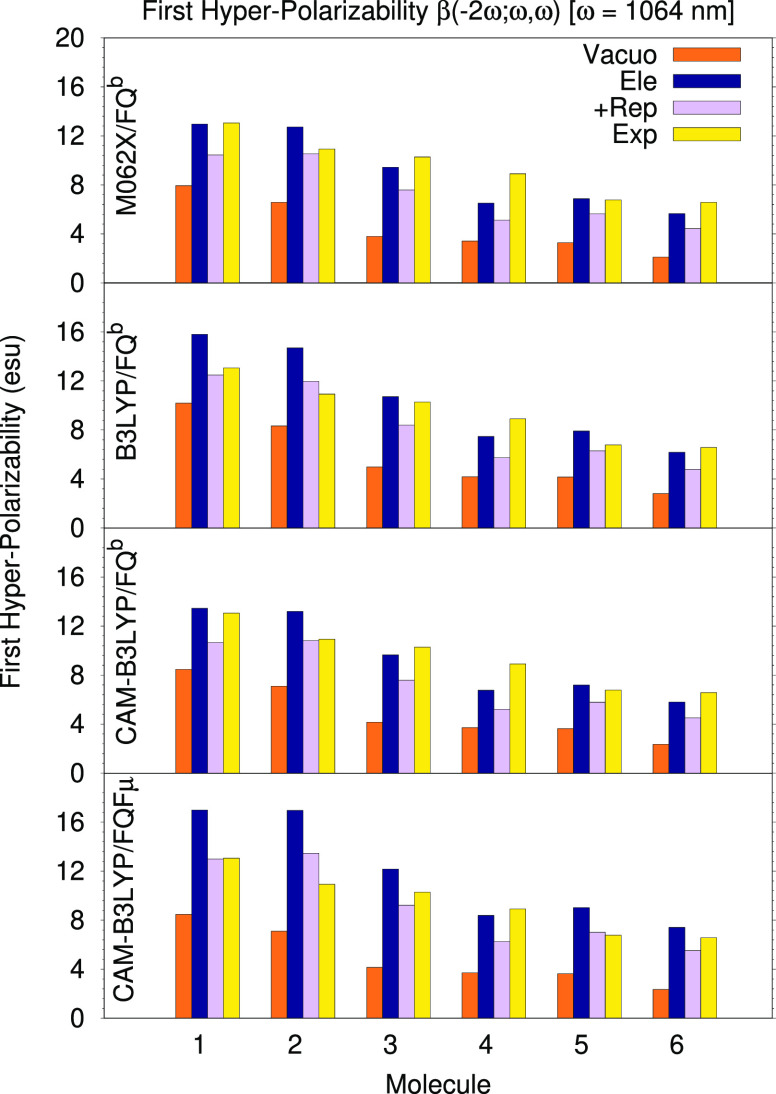
Dynamic hyperpolarizabilities of molecules **1**–**6** evaluated at 1064 nm in vacuo and in solution (with and without repulsion effects) evaluated with different functionals and solvation models. Experimental data from Ray et al.^71^

Figure 8 reports the average values of the dynamical hyperpolarizabilities *β*( – 2*ω*; *ω*, *ω*) computed with three functionals, with and without quantum repulsion, as well as the experimental values obtained by means of hyper-Rayleigh scattering (HRS) measurements in ref (71). Numerical values for the solvated system are also reported in Table 1 for an easier reading.

**Table 1:** CAM-B3LYP, B3LYP, and M06-2X with QM/FQ*^b^* Parameters, with and without Repulsion *β*( – 2*ω*; *ω*, *ω*) (± Standard Errors, Values in esu)

*β*( – 2*ω*; *ω*, *ω*)												
	CAM-B3LYP			B3LYP			M06-2X				CAM-B3LYP/FQFμ	
	w/o rep	rep	vacuum	w/o rep	rep	vacuum	w/o rep	rep	vacuum	EXP^71^	w/o rep	rep
**1**	13.45 ± 0.26	10.65 ± 0.19	8.47	15.81 ± 0.33	12.49 ± 0.25	10.19	12.96 ± 0.26	10.44 ± 0.21	7.94	13.06	16.99 ± 0.34	12.99 ± 0.24
**2**	13.19 ± 0.16	10.81 ± 0.12	7.10	14.70 ± 0.17	11.97 ± 0.13	8.33	12.72 ± 0.16	10.54 ± 0.12	6.58	10.93	16.96 ± 0.23	13.46 ± 0.16
**3**	9.65 ± 0.14	7.58 ± 0.10	4.15	10.72 ± 0.16	8.40 ± 0.11	4.98	9.44 ± 0.14	7.58 ± 0.11	3.80	10.28	12.17 ± 0.18	9.22 ± 0.12
**4**	6.76 ± 0.10	5.21 ± 0.07	3.71	7.47 ± 0.12	5.73 ± 0.08	4.18	6.51 ± 0.10	5.13 ± 0.08	3.42	8.91	8.39 ± 0.13	6.25 ± 0.09
**5**	7.19 ± 0.10	5.78 ± 0.07	3.63	7.91 ± 0.11	6.30 ± 0.07	4.16	6.87 ± 0.09	5.64 ± 0.08	3.28	6.78	9.02 ± 0.12	7.01 ± 0.08
**6**	5.80 ± 0.09	4.51 ± 0.06	2.35	6.18 ± 0.10	4.78 ±0.07	2.81	5.65 ± 0.09	4.43 ± 0.06	2.10	6.57	7.41 ± 0.11	5.52 ± 0.08

Comparing gas-phase values with electrostatics-only solvated values (whether obtained with the FQ*^b^* or the FQFμ model), we see that, in some cases, the computed property can even double in value. However, as was observed for polarizabilities, repulsion has a the opposite effect; however, in this case, the decrease is much more pronounced, being on average about 20%, compared to 8% of simple polarizabilities. This result emphasizes the important role played by repulsion effects in determining high-order electric properties of systems in the condensed phase and suggests that any quantitative calculation of such properties for systems in solution should not neglect them. The final result is the product of a delicate balance between these opposing effects, though all values in solution are larger than the corresponding gas-phase results. These results speak to a large extent about the fact that one must be careful when evaluating the performance of any solvation model that only accounts for electrostatics, such as plain QM/FQ*^b^* or the popular polarizable continuum model (PCM). Results that are closer to the experiment might be achieved by lowering the solvent’s polarization through a careful parametrization of the method, such as an increase in the dimension of the PCM cavity or tinkering with the FQ parameters, though this would only be so because of a fruitful and artificial error cancellation. The compensation between electrostatic and non-electrostatic forces, however, is not consistent across different molecular properties (as can be seen by simply comparing the data in Figure 6 for polarizabilities and Figure 8 for hyperpolarizabilities); therefore, error cancellation will not work for all properties leading to a systematic error in the results.

Finally, we can compare our calculations experimental data. We see that, in some cases, the QM/FQ*^b^*+rep model apparently leads to a greater error compared to the simpler QM/FQ*^b^* purely electrostatic model. This is observed for all systems except for molecule **2** when using the CAM-B3LYP and M06-2X functionals, though not in the case of the B3LYP functional where molecule **1** is also an exception. The inclusion of polarizable dipoles in the solvent’s description leads to a further increase in the computed values, as observed in the case of static and dynamics polarizabilities and, with the exception of molecule **2**, produces values that are much closer to their experimental counterparts if repulsion is also included.

## Conclusions and Perspectives

In this paper, we have presented a computational study of polarizabilities and hyperpolarizabilities of molecules in aqueous solutions. We dissected the solute–solvent interaction into its electrostatic and non-electrostatic components and then compared computed results with experimental findings to assess the role of each interaction. As a solvation model, we employed our recently developed polarizable QM/MM method based on fluctuating charges and dipoles (FQ and FQFμ) enriched by solute–solvent repulsion effects to the calculation of polarizabilities and hyperpolarizabilities of organic molecules in water. By dissecting the magnitude and role of each component of the solvation phenomenon as it applies to the set of studied systems, we showed that QM/FQ and QM/FQFμ models for solvation electrostatics can be combined with our recently implemented quantum repulsion model to successfully calculate linear and non-linear electric response properties of systems in solution in a “focused” solvation model paradigm. This is possible owing to the model’s ability to be extended to high-order properties through the propagation of the solute–solvent interaction terms at all orders of the QM response functions. Our results show that all of the different effects we considered contribute to the computed value in similar measures, meaning that none of them can be safely neglected. In particular, the modeling of electrostatic effects with the FQ method leads to an expected increase in the computed polarizability values compared to the isolated molecule, which is further intensified by the addition of polarizable dipoles in the solute’s description. Repulsion has an effect that is similar in magnitude but opposite in sign; therefore, the evaluation of such properties is the result of a delicate balance between all these contrasting forces, which in principle must all be included in the model and treated as accurately as possible. While numerically decent results might be obtained by neglecting repulsion altogether and tinkering with the magnitude of the solvent polarization (or removing it altogether as is done with standard non-polarizable QM/MM methods), this approach should not be regarded as “safe” or generally transferable to a wide array of systems for which the one effect or the other may dominate. Our results therefore underline the complexity of the forces at play within a water solution, which, far from being simply a highly polar substance with the ability to form hydrogen bonds, can influence a solute’s properties through effects such as quantum repulsion and electronic polarizability, which can be almost as important as the presence of hydrogen bonds themselves.

This work’s results notwithstanding, much work remains to be done in this field. To fully appreciate the improvements offered by such refined models over more standard methodologies, a wide benchmark over a wider set of systems and solvents should be performed to estimate the expected error of the model for a given functional and basis set. In addition, a much wider array of response properties, particularly those involving a magnetic or mixed electric and magnetic response such as nuclear magnetic shields or optical rotatory dispersion should be investigated to fully appreciate the power of the method. Finally, one type of solvent effect that was neglected in this work is that, due to electron dispersion, while it has been shown to be negligible in the case of water,^47,60,62,74^ it can be expected to be particularly relevant for solvents such as benzene, and models to include this effect in the evaluation of high-order response properties in an efficient manner should be investigated and will be the object of future work.

## Supporting Information Available

Convergence of QM/MM polarizabilities and first hyperpolarizabilities as a function of the snapshots’ number; data related to Figures 5, 6, and 8; and plotted density matrix derivatives (PDF)
