# Supplementary material for: Calculation of Linear and Non-linear Electric Response Properties of Systems in Aqueous Solution: A Polarizable Quantum/Classical Approach with Quantum Repulsion Effects
Source: J Chem Theory Comput. 2020 Oct 15;16(11):6993–7004. doi: 10.1021/acs.jctc.0c00674 (PMC8015238; doi:10.1021/acs.jctc.0c00674)
Supplement: Supplementary file 1 — ct0c00674_si_001.pdf [file ct0c00674_si_001.pdf]

Supporting Information: Calculation of linear and non-linear electric response properties of systems in aqueous solution: a polarizable QM/MM approach with quantum repulsion effects

Gioia Marrazzini,[†] Tommaso Giovannini,[‡] Franco Egidi,[*,†] and Chiara Cappelli[*,†]

†*Scuola Normale Superiore, Piazza dei Cavalieri 7, 56126 Pisa, Italy.*

‡*Department of Chemistry, Norwegian University of Science and Technology, 7491 Trondheim, Norway*

E-mail: franco.egidi@sns.it; chiara.cappelli@sns.it

S0.1 Convergence of QM/MM calculations

The quality of QM/MM results rests on the assumption that the entire solute-solvent configuration space has been sampled by the MD and the number of extracted snapshots is sufficient. Final results are obtained by averaging the computed values across the whole set of snapshots. In Fig. S1 we report the average of each property (static polarizability $\alpha(0;0)$, dynamic polarizability $\alpha(-\omega;\omega)$, and dynamic hyperpolarizability $\beta(-2\omega;\omega,\omega)$) as a function of the number of snapshots, obtained at the CAM-B3LYP/FQ^b level of theory. We can see that for most molecules convergence is achieved within the first 100 snapshots. The only exception to this seems to be molecule 1, for which we observe further oscillation past the 100 snapshot mark, and for which 200 snapshots are necessary to achieve convergence. Nonetheless, oscillations around the average values remain quite small and most of

the sampling is still achieved within the first 100 snapshots. These conclusions hold for all three properties analyzed, both with and without repulsion effects.

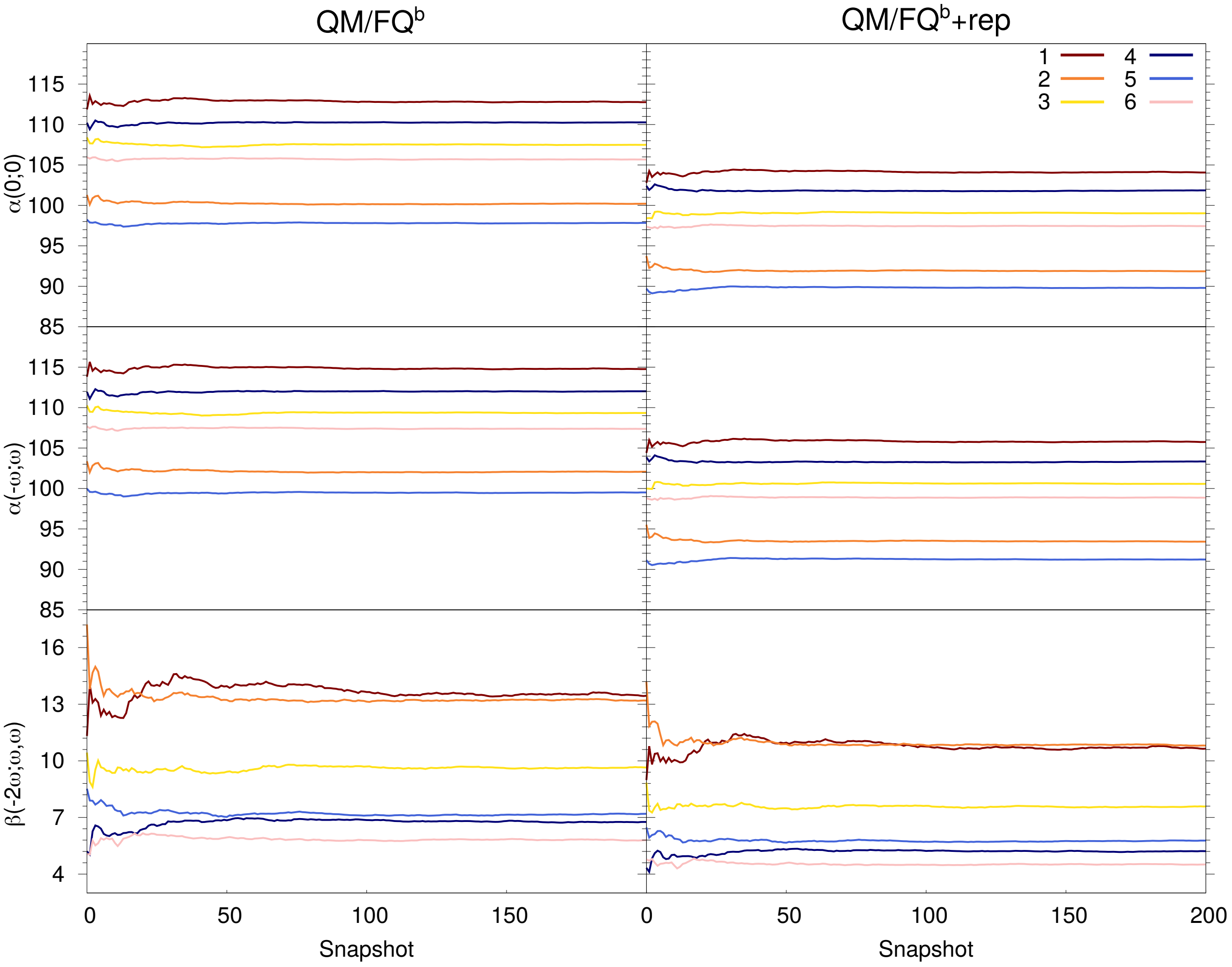

Figure S1: Average values of $\alpha(0;0)$, $\alpha(-\omega;\omega)$ and $\beta(-2\omega;\omega,\omega)$ as functions of the number of snapshots exctracted from MD for all the molecules considered using the CAM-B3LYP/FQ^b method. On the left, snapshots without repulsion, while, on the right, snapshots with repulsion.

S0.2 Tabulated QM/MM polarizabilities and hyperpolarizabilities

We report here tabulated numerical values corresponding to those presented in figures within the main manuscript. QM/FQ and QM/FQFμ data also come with an error bar given by the variance of the computed property across the snapshots extracted from the classical MD. Gas-phase values, by contrast, do not have error bars because they are just single-point calculations peformed on an optimized molecular geometry. Table S1 shows the static and dynamic polarizability of the six molecular systems studied, both in water in vacuo, with and without repulsion effects, with three different functionals. Table S2 shows how the same values, restricted to the CAM-B3LYP functional, change with changing solvation model, i.e. QM/FQ^a, QM/FQ^b, or QM/FQFμ. Tables S2 reports dynamical hyperpolarizabilities, evaluated with the same three functionals, in vacuo and in water, the latter modeled with the QM/FQ^b method with and without repulsion effect. CAM-B3LYP/FQFμ values are also reported.

Table S1: Static and dynamic polarizabilities computed with the QM/FQ method with and without repulsion effects in cm^3/mol.

	α(0;0)								
	**CAM-B3LYP**			**B3LYP**			**M06-2X**		
	**w/o rep**	**rep**	**vacuum**	**w/o rep**	**rep**	**vacuum**	**w/o rep**	**rep**	**vacuum**
**1**	10.06±0.011	9.29±0.009	9.30	10.32±0.009	9.50±0.011	9.55	9.93±0.011	9.22±0.009	9.17
**2**	8.94 ±0.008	8.24±0.006	8.07	9.16 ±0.006	8.38±0.007	8.27	8.81±0.008	8.13±0.006	7.95
**3**	9.59 ±0.006	8.84±0.005	8.76	9.81 ±0.005	9.01±0.006	8.96	9.48±0.007	8.79±0.005	8.65
**4**	9.84 ±0.007	9.09±0.006	9.16	10.06±0.006	9.27±0.006	9.38	9.71±0.007	9.02±0.005	9.03
**5**	8.73 ±0.006	8.01±0.004	7.97	8.92 ±0.004	8.17±0.005	8.15	8.61±0.006	7.94±0.004	7.85
**6**	9.43 ±0.005	8.70±0.004	8.68	9.63 ±0.004	8.86±0.004	8.87	9.32±0.005	8.64±0.004	8.58

	α(-ω;ω)								
	**CAM-B3LYP**			**B3LYP**			**M06-2X**		
	**w/o rep**	**rep**	**vacuum**	**w/o rep**	**rep**	**vacuum**	**w/o rep**	**rep**	**vacuum**
**1**	10.24±0.012	9.44±0.009	9.44	10.52±0.013	9.67±0.012	9.70	10.10±0.012	9.36±0.010	9.30
**2**	9.11 ±0.009	8.34±0.007	8.19	9.35 ±0.010	8.53±0.008	8.40	8.97 ±0.009	8.26±0.007	8.07
**3**	9.76 ±0.007	8.98±0.006	8.88	9.99 ±0.008	9.17±0.006	9.09	9.64 ±0.007	8.93±0.006	8.77
**4**	10.00±0.008	9.22±0.006	9.28	10.24±0.008	9.42±0.007	9.51	9.86 ±0.008	9.15±0.006	9.15
**5**	8.88 ±0.006	8.14±0.005	8.08	9.09 ±0.006	8.31±0.005	8.27	8.75 ±0.006	8.07±0.005	7.96
**6**	9.58 ±0.006	8.82±0.005	8.80	9.79 ±0.006	8.99±0.005	8.99	9.47 ±0.005	8.77±0.004	8.69

Table S2: Static and dynamic polarizabilities, in cm^3/mol, computed with the CAM-B3LYP/FQ method with and without repulsion effects, using either Rick[1] or QM/FQ^b[2] parameters for the electrostatic part and the vacuum data.

α**(0;0)**

	**Rick**		**QM/FQ^b**		**QM/FQFμ**		**Vacuum**
	**w/o rep**	**rep**	**w/o rep**	**rep**	**w/o rep**	**rep**	
**1**	9.87	9.12	10.06	9.29	10.54	9.68	9.30
**2**	8.73	8.01	8.94	8.24	9.41	8.58	8.07
**3**	9.40	8.67	9.59	8.84	10.04	9.20	8.76
**4**	9.69	8.95	9.84	9.09	10.28	9.45	9.16
**5**	8.57	7.87	8.73	8.01	9.16	8.37	7.97
**6**	9.27	8.55	9.43	8.70	9.86	9.05	8.68

$\alpha(-\omega;\omega)$

	**Rick**		**QM/FQ^b**		**CAM-B3LYP/QM/FQFμ**		**Vacuum**
	**w/o rep**	**rep**	**w/o rep**	**rep**	**w/o rep**	**rep**	
**1**	10.04	9.25	10.24	9.44	10.74	9.84	9.44
**2**	8.88	8.14	9.11	8.34	9.60	8.74	8.19
**3**	9.56	8.80	9.76	8.98	10.22	9.35	8.88
**4**	9.83	9.08	10.00	9.22	10.46	9.60	9.28
**5**	8.71	7.99	8.88	8.14	9.33	8.51	8.08
**6**	9.41	8.67	9.58	8.82	10.03	9.19	8.80

Table S3: CAM-B3LYP, B3LYP and M06-2X with QM/FQ^b parameters, with and without repulsion β(-2ω;ω,ω) (± standard errors, values in esu)

	$\beta(-2\omega;\omega,\omega)$											
	**CAM-B3LYP**			**B3LYP**			**M06-2X**			**EXP[3]**	**CAM-B3LYP/FQFμ**	
	**w/o rep**	**rep**	**vacuum**	**w/o rep**	**rep**	**vacuum**	**w/o rep**	**rep**	**vacuum**		**w/o rep**	**rep**
**1**	13.45±0.26	10.65±0.19	8.47	15.81±0.33	12.49±0.25	10.19	12.96±0.26	10.44±0.21	7.94	13.06	16.99±0.34	12.99±0.24
**2**	13.19±0.16	10.81±0.12	7.10	14.70±0.17	11.97±0.13	8.33	12.72±0.16	10.54±0.12	6.58	10.93	16.96±0.23	13.46±0.16
**3**	9.65 ±0.14	7.58 ±0.10	4.15	10.72±0.16	8.40 ±0.11	4.98	9.44 ±0.14	7.58 ±0.11	3.80	10.28	12.17±0.18	9.22 ±0.12
**4**	6.76 ±0.10	5.21 ±0.07	3.71	7.47 ±0.12	5.73 ±0.08	4.18	6.51 ±0.10	5.13 ±0.08	3.42	8.91	8.39 ±0.13	6.25 ±0.09
**5**	7.19 ±0.10	5.78 ±0.07	3.63	7.91 ±0.11	6.30 ±0.07	4.16	6.87 ±0.09	5.64 ±0.08	3.28	6.78	9.02 ±0.12	7.01 ±0.08
**6**	5.80 ±0.09	4.51 ±0.06	2.35	6.18 ±0.10	4.78 ±0.07	2.81	5.65 ±0.09	4.43 ±0.06	2.10	6.57	7.41 ±0.11	5.52 ±0.08

S0.3 Plotted density matrix derivatives

We report here a list of the electric field derivatives of the density matrix for a randomly selected snapshot of the molecule **1** (see main article text for more details) with and without the inclusion of repulsion effects. The density matrices are in the molecular orbital basis and shown in figures S2 and S3. It can be observed that derivatives along the x direction are less affected by repulsion compared to the other two direction. In addition, off-diagonal derivatives are also less influenced by repulsion.

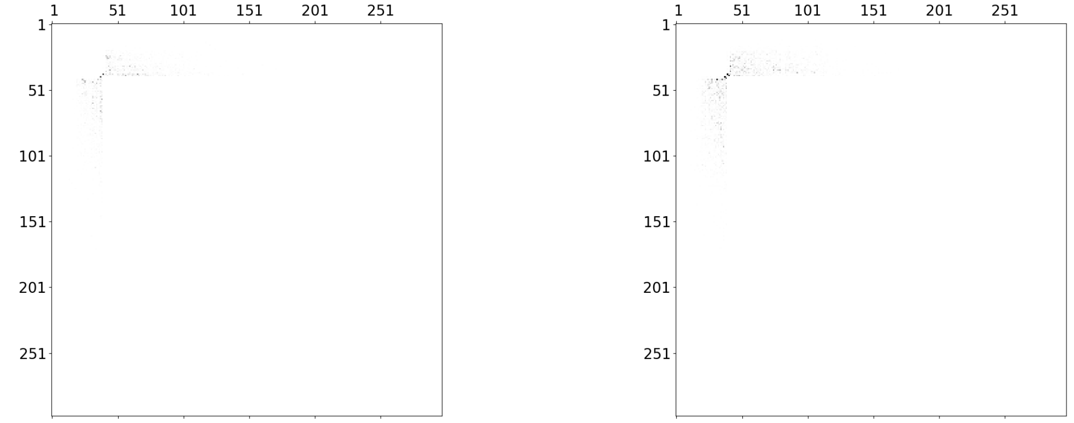

$\mathbf{P}^{(1)}_{\mathrm{rep-norep}}(x)$ $\mathbf{P}^{(1)}_{\mathrm{rep-norep}}(y)$

Figure S2: Difference between the density matrix first derivatives with and without Pauli repulsion of a randomly snapshot of molecule **1** extracted from MD simulation. Derivatives are taken with respect to the components of the electric field indicated in parenthesis.

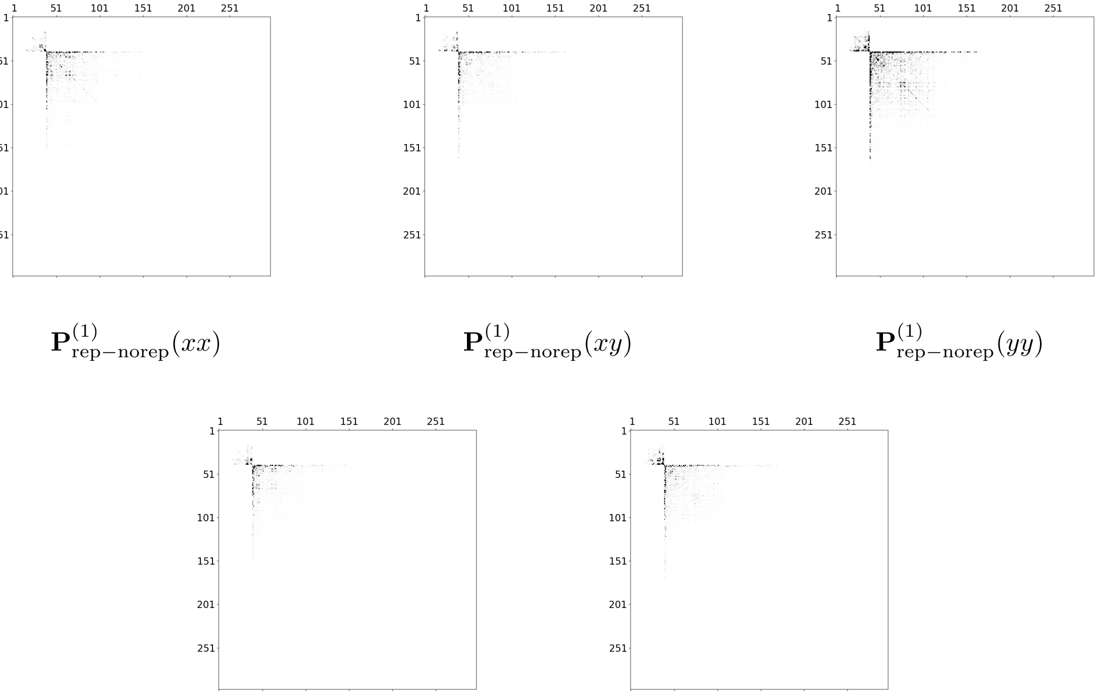

$\mathbf{P}^{(1)}_{\mathrm{rep-norep}}(xx)$ $\mathbf{P}^{(1)}_{\mathrm{rep-norep}}(xy)$ $\mathbf{P}^{(1)}_{\mathrm{rep-norep}}(yy)$

$\mathbf{P}^{(1)}_{\mathrm{rep-norep}}(xz)$ $\mathbf{P}^{(1)}_{\mathrm{rep-norep}}(yz)$

Figure S3: Difference between the density matrix second derivatives with and without Pauli repulsion of a randomly snapshot of molecule **1** extracted from MD simulation. Derivatives are taken with respect to the components of the electric field indicated in parenthesis.

References

(1) Rick, S. W.; Stuart, S. J.; Berne, B. J. Dynamical fluctuating charge force fields: Application to liquid water. *The Journal of chemical physics* **1994**, *101*, 6141–6156.

(2) Giovannini, T.; Lafiosca, P.; Chandramouli, B.; Barone, V.; Cappelli, C. Effective yet reliable computation of hyperfine coupling constants in solution by a QM/MM approach: Interplay between electrostatics and non-electrostatic effects. *The Journal of chemical physics* **2019**, *150*, 124102.

(3) Ray, P. C.; Das, P. K.; Ramasesha, S. A comparative study of first hyperpolarizabilities of the acidic and basic forms of weak organic acids in water. *The Journal of chemical physics* **1996**, *105*, 9633–9639.
